# From hype to reality: data science enabling personalized medicine

**DOI:** 10.1186/s12916-018-1122-7

**Published:** 2018-08-27

**Authors:** Holger Fröhlich, Rudi Balling, Niko Beerenwinkel, Oliver Kohlbacher, Santosh Kumar, Thomas Lengauer, Marloes H. Maathuis, Yves Moreau, Susan A. Murphy, Teresa M. Przytycka, Michael Rebhan, Hannes Röst, Andreas Schuppert, Matthias Schwab, Rainer Spang, Daniel Stekhoven, Jimeng Sun, Andreas Weber, Daniel Ziemek, Blaz Zupan

**Affiliations:** 1 UCB Biosciences GmbH, Alfred-Nobel-Str. Str. 10, 40789 Monheim, Germany; 2 University of Luxembourg, 6 avenue du Swing, 4367 Belvaux, Luxembourg; 3 Department of Biosciences and Engineering, ETH Zurich, Mattenstr. 26, 4058 Basel, Switzerland; 4 University of Tübingen, WSI/ZBIT, Sand 14, 72076 Tübingen, Germany; 5 Department of Computer Science, University of Memphis, 2222 Dunn Hall, Memphis, TN 38152 USA; 6 Max-Planck-Institute for Informatics, 66123 Saarbrücken, Germany; 7 ETH Zurich, Seminar für Statistik, Rämistrasse 101, 8092 Zurich, Switzerland; 8 University of Leuven, ESAT, Kasteelpark Arenberg 10, 3001 Leuven, Belgium; 9 Harvard University, Science Center 400 Suite, Oxford Street, Cambridge, MA 02138-2901 USA; 10 National Center of Biotechnology Information, National Institute of Health, 8600 Rockville Pike, Bethesda, MD 20894-6075 USA; 11 Novartis Institutes for Biomedical Research, 4056 Basel, Switzerland; 12 Donnelly Centre for Cellular and Biomolecular Research, University of Toronto, 160 College Street, Toronto, ON M5S 3E1 Canada; 13 RWTH Aachen, Joint Research Center for Computational Biomedicine, Pauwelsstrasse 19, 52074 Aachen, Germany; 14 Dr. Margarete Fischer-Bosch Institute of Clinical Pharmacology, Aucherbachstrasse 112, 70376 Stuttgart, Germany; 15 University of Regensburg, Institute of Functional Genomics, Am BioPark 9, 93053 Regensburg, Germany; 16 ETH Zurich, NEXUS Personalized Health Technol., Otto-Stern-Weg 7, 8093 Zurich, Switzerland; 17 Georgia Tech University, 801 Atlantic Drive, Atlanta, GA 30332-0280 USA; 18 Institute for Computer Science, University of Bonn, Endenicher Allee 19a, 53115 Bonn, Germany; 19 Pfizer, Worldwide Research and Development, Linkstraße 10, 10785 Berlin, Germany; 20 Faculty of Computer and Information Science, University of Ljubljana, Večna pot 113, SI-1000 Ljubljana, Slovenia; 21 University of Bonn, Bonn-Aachen International Center for IT, Endenicher Allee 19c, 53115 Bonn, Germany; 22 Max Planck Institute for Developmental Biology, Max-Planck-Ring 5, 72076 Tübingen, Germany; 23 Quantitative Biology Center, University of Tübingen, Auf der Morgenstelle 8, 72076 Tübingen, Germany; 24 Institute for Translational Bioinformatics, University Medical Center Tübingen, Sand 14, 72076 Tübingen, Germany; 25 University of Tübingen, Departments of Clinical Pharmacology and of Pharmacy and Biochemistry, Tübingen, Germany

**Keywords:** Personalized medicine, Precision medicine, Stratified medicine, P4 medicine, Machine learning, Artificial intelligence, Big data, Biomarkers

## Abstract

**Background:**

Personalized, precision, P4, or stratified medicine is understood as a medical approach in which patients are stratified based on their disease subtype, risk, prognosis, or treatment response using specialized diagnostic tests. The key idea is to base medical decisions on individual patient characteristics, including molecular and behavioral biomarkers, rather than on population averages. Personalized medicine is deeply connected to and dependent on data science, specifically machine learning (often named Artificial Intelligence in the mainstream media). While during recent years there has been a lot of enthusiasm about the potential of ‘big data’ and machine learning-based solutions, there exist only few examples that impact current clinical practice. The lack of impact on clinical practice can largely be attributed to insufficient performance of predictive models, difficulties to interpret complex model predictions, and lack of validation via prospective clinical trials that demonstrate a clear benefit compared to the standard of care. In this paper, we review the potential of state-of-the-art data science approaches for personalized medicine, discuss open challenges, and highlight directions that may help to overcome them in the future.

**Conclusions:**

There is a need for an interdisciplinary effort, including data scientists, physicians, patient advocates, regulatory agencies, and health insurance organizations. Partially unrealistic expectations and concerns about data science-based solutions need to be better managed. In parallel, computational methods must advance more to provide direct benefit to clinical practice.

## Background

Personalized, precision, P4, or stratified medicine is understood as a medical approach in which patients are stratified based on their disease subtype, risk, prognosis, or treatment response using specialized diagnostic tests [1]. In many publications, the terms mentioned above are used interchangeably, although some authors make further distinctions between them to highlight particular nuances. The key idea is to base medical decisions on individual patient characteristics (including biomarkers) rather than on averages over a whole population. In agreement with the US Food and Drug Administration (FDA; https://www.fda.gov/ucm/groups/fdagov-public/@fdagov-drugs-gen/documents/document/ucm533161.pdf), we herein use the term biomarker for any measurable quantity or score that can be used as a basis to stratify patients (e.g., genomic alterations, molecular markers, disease severity scores, lifestyle characteristics, etc). The advantages of personalized medicine (summarized in [2, 3]) are widely considered to be (1) better medication effectiveness, since treatments are tailored to patient characteristics, e.g., genetic profile; (2) reduction of adverse event risks through avoidance of therapies showing no clear positive effect on the disease, while at the same time exhibiting (partially unavoidable) negative side effects; (3) lower healthcare costs as a consequence of optimized and effective use of therapies; (4) early disease diagnosis and prevention by using molecular and non-molecular biomarkers; (5) improved disease management with the help of wearable sensors and mobile health applications; and (6) smarter design of clinical trials due to selection of likely responders at baseline.

**Fig. 1:**
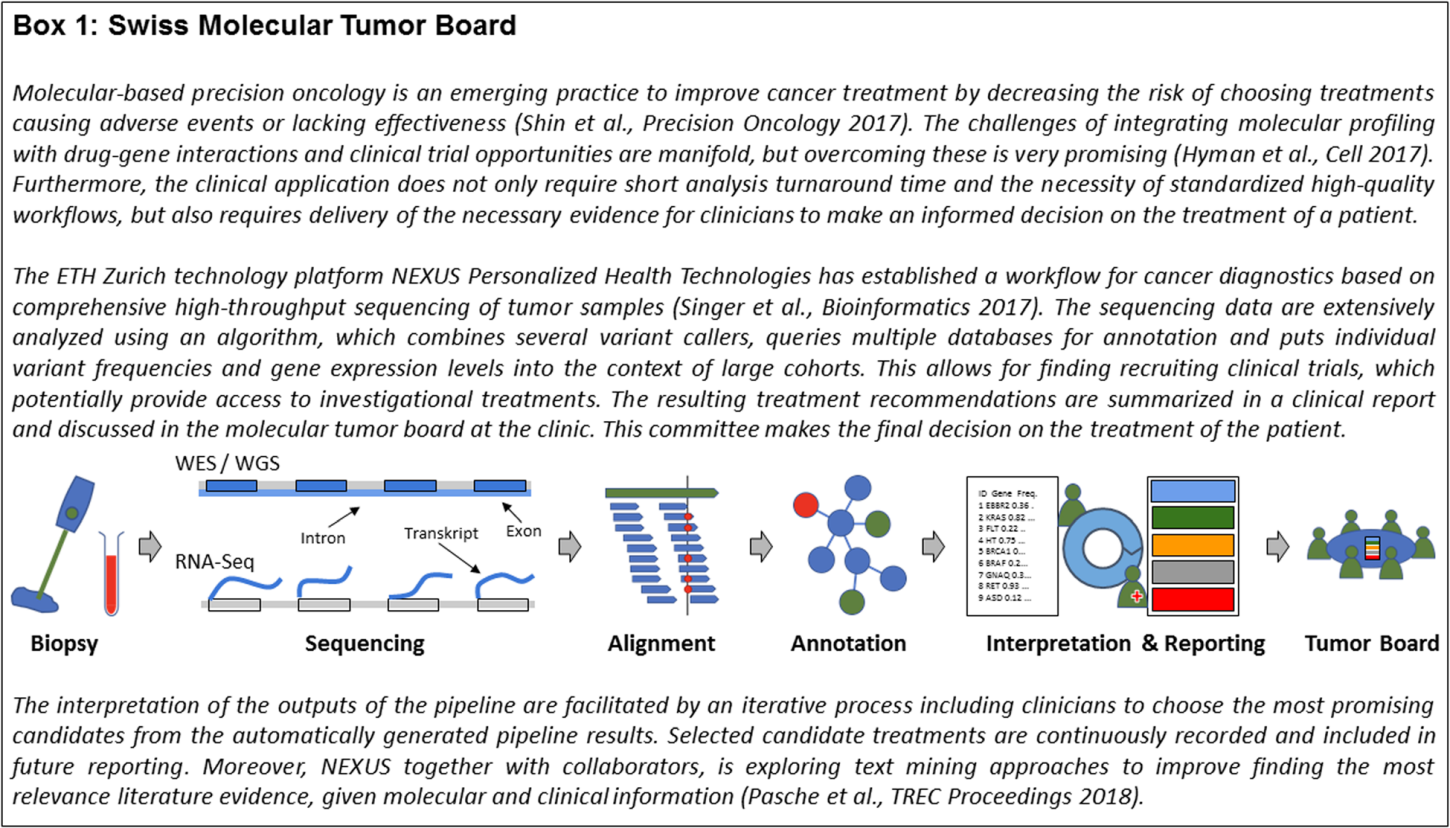
The Swiss molecular tumor board as an example of individualized, biomarker-based medical decisions in clinical practice

At present, personalized medicine is only an emerging reality. Molecular tumor boards at hospitals are probably furthest in realizing the promises of personalized medicine in clinical practice (Fig. 1). At the same time, this example already demonstrates a strong dependency of personalized medicine on computational solutions. Herein, we first explain, how modern approaches from data science, and specifically machine learning, are now beginning to impact personalized medicine. However, the way in which machine learning (often used interchangeably with the term Artificial Intelligence) is presented in the mainstream media often constitutes a hype, which must be contrasted with reality. We identify several challenges that currently constitute hurdles for realizing machine learning-based solutions more broadly in clinical practice. We discuss these challenges together with the existing potential of data science for personalized medicine. Finally, we highlight directions for future development.

## Data science increasingly impacts personalized medicine

To date, the FDA has listed more than 160 (mostly genomic) pharmacogenomic biomarkers (https://www.fda.gov/Drugs/ScienceResearch/ucm572698.htm) and biomarker signatures (oncology: 33.5%; neurology: 6.1%) that have been approved for stratifying patients for drug response. For example, the anti-cancer drug trastuzumab (Herceptin^®^) can only be administered if the HER2/neu receptor is overexpressed because the drug interferes with this receptor. Personalized medicine is nowadays thus tightly connected with genomics. However, genomics and other biological high throughput data (transcriptomics, epigenomics, proteomics, metabolomics) are by no means the only source of data employed in the personalized medicine field. Other relevant data include, for example, bio-images (e.g., MRT and CT scans), electronic medical records (EMRs) [4], health claims data from insurance companies [5], and data from wearable sensors and mobile health applications [6].

It is important to mention that, in many cases, it is impossible to identify a single stratification factor or biomarker for patient populations. This is because many diseases (including cancer and various neurological and immunological diseases) are complex and affect a multitude of biological sub-systems. Accordingly, drugs for treating these diseases often target multiple proteins and associated biological processes [7]. In general, clinical drug response is highly multi-faceted and dependent on a combination of patient intrinsic (e.g., genomic, age, sex, co-medications, liver function) and extrinsic (e.g., alcohol consumption, diet, sunlight exposure) factors [8]. In conclusion, single-analyte biomarker patient stratification, such as in the Herceptin® example, is only possible in special cases.

**Fig. 2:**
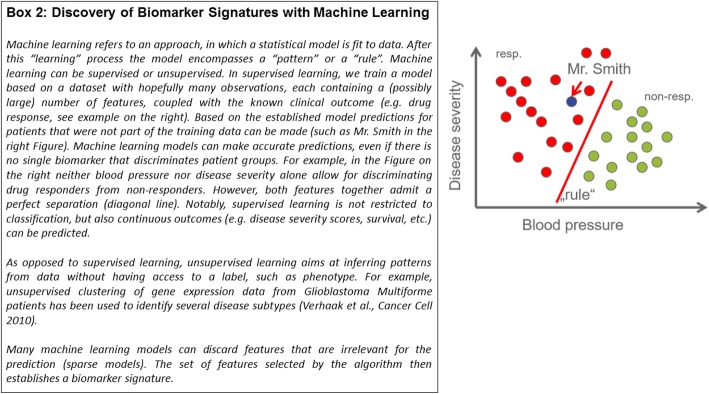
Discovery of biomarker signatures with machine learning

**Fig. 3:**
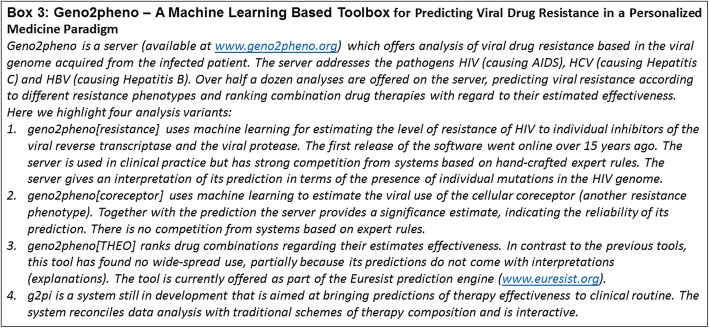
Geno2pheno - a machine learning based toolbox for predicting viral drug resistance in a personalized medicine paradigm

An alternative to single-analyte biomarkers are multi-analyte signatures derived from complex, high-throughput data, which allow patient characterization in a much more holistic manner than single biomarkers. Identifying marker signatures is difficult and requires state-of-the-art approaches offered by data science. Specifically, multivariate stratification algorithms using techniques from the area of Artificial Intelligence (including machine learning) play an increasingly important role (Fig. 2). A highly-cited example is MammaPrint™, a prognostic test for breast cancer based on a 70-gene signature [9], which was approved by the FDA in 2007. MammaPrint™ produces a score from the weighted average of 70 measured genes, which is predictive for the development of distant metastases. The clinical utility of the addition of the MammaPrint™ signature compared to standard clinicopathological criteria has been recently shown in selecting patients for adjuvant chemotherapy [10]. Other examples are *Geno2pheno* [11, 12], which is a computational tool used in clinical practice to estimate the resistance of HIV to an individual drug and to combinatorial therapies based on viral genotype (Fig. 3), and a gene signature (S3 score) for prediction of prognosis in patients with clear cell renal cell carcinoma [13].

Driven by the increasing availability of large datasets, there is a growing interest into such data science-driven solutions. Specifically, ‘deep learning’ techniques have received a lot of attention, for example, in radiology [14, 15], histology [16] and, more recently, in the area of personalized medicine [17–20]. Some of these algorithms have been reported to achieve above-human diagnostic performance in certain cases [21]. Large commercial players now entering the field underline the widely perceived potential for machine learning-based solutions within personalized medicine (https://www.techemergence.com/machine-learning-in-pharma-medicine/, http://bigthink.com/ideafeed/for-new-era-of-personalized-medicine-google-to-store-individual-genomes-in-the-cloud, http://medicalfuturist.com/innovative-healthcare-companies/).

## The data science and AI hype contrasts with reality

### The mainstream media perception

From the previous discussion one might get the impression that enabling personalized medicine is mainly a matter of availability of ‘big data’, sufficient computing power, and modern deep-learning techniques. Indeed, this perception is portrayed in many mainstream publications, read by decision-makers in politics and industry (https://www.fool.com/investing/2017/09/21/3-ways-ai-is-changing-medicine.aspx, http://www.healthcareitnews.com/slideshow/how-ai-transforming-healthcare-and-solving-problems-2017?page=1, http://medicalfuturist.com/artificial-intelligence-will-redesign-healthcare/). In that context, some authors have even claimed the end of classical, hypothesis-driven science and stated that, in the future, all novel insights would come from an algorithmic analysis of large datasets (https://www.wired.com/2008/06/pb-theory/).

Such statements are overly optimistic and overlook several important aspects, which we discuss below.

### Challenge 1: insufficient prediction performance for clinical practice

Machine learning methods capture and mathematically describe a (complex) signal that is present in a dataset. Their success does not only depend on the number of (patient) samples, but also on the signal-to-noise ratio. Indeed, separation of true signal from technical noise is still one of the key challenges in big data analysis [22] and one of the key aspects of any computational model. More generally, the prediction performance of any machine learning model is limited per se by the descriptive power of the employed data with respect to the clinical endpoint of interest. For example, EMRs are longitudinal, but largely phenotypic. Thus, molecular phenomena (e.g., non-common genomic variants) that might be relevant to stratifying patients are not sufficiently represented in the data. On the other hand, genomic data is mostly static (at least in non-cancerous tissues) and misses potentially important longitudinal clinical information. For each prediction problem, it is therefore critical to identify and combine the right data modalities that could contain parts of the relevant signal when starting to build machine learning models. Shortcomings can result in loss of prediction performance. Many machine learning models developed for personalized medicine do not have a predictive power close to the high (and potentially unrealistic) expectations of clinicians. Some of the reasons are as follows:

- The relationships of patient-specific characteristics to clinically relevant endpoints are highly complex and non-linear, often varying over time and, as mentioned before, typically not well described by one data instance alone. Furthermore, discriminating relevant from irrelevant patient-specific features remains a challenge, specifically in the field of biological high throughput (omics) data.
- It is challenging to obtain a sufficiently large patient cohort with well-defined phenotypes for training and testing models due to cost and time constraints.
- Many data (e.g., most omics data) are very noisy. There are two sources of this noise. One is technical measurement error (undesired), the other is biological variation (highly informative). We have no good methods for discriminating between these two kinds of noise.
- It can be challenging to quantitatively and objectively define clinical outcomes (e.g., in neurology, immunology, and psychology). This can lead to highly subjective and physician-dependent variations.
- Clinical outcomes may vary over time and be partially influenced by factors that are not patient intrinsic and thus hard to capture (e.g., social and environmental influences).
- A further factor impacting prediction performance is the careful choice of patient samples. Machine learning models are typically sensitive to selection biases, i.e., under- or over-represented specific patient subgroups in the training cohort, and there are currently under-explored ethical considerations at play as well. For example, over- or under-representation of certain ethnicities could result into a ‘racist’ prediction model [23]. A proper and careful design of the training set is necessary to ensure that it is representative for the population of patients in the intended application phase of the model in clinical practice.

### Challenge 2: difficulties in interpretation

The scientific approach, which has been successfully established since the times of Galileo Galilei in the sixteenth century, always encompasses an ongoing process of hypothesis formulation and experimental validation [24]. While machine learning techniques can detect complex patterns in large data and provide accurate predictions, in general – we will discuss details later – they are unable to provide a deeper theoretical, mechanistic, or causal understanding of an observed phenomenon. Data science and AI thus do not replace classical, hypothesis-driven research. One reason is that machine learning models typically only capture statistical dependencies, such as correlation, from data. However, correlation does not imply causation. This is reflected by the fact that a multitude of biomarker signatures yielding similar prediction performance can be constructed to separate the same patient groups [25]. Even if an acceptable prediction performance can be achieved, the lack of a clear causal or mechanistic interpretation of machine learning models can hinder acceptance of data science-based solutions by physicians.

### Challenge 3: insufficient validation for clinical practice

It is important to emphasize that establishing any algorithm for patient stratification in clinical practice requires rigorous validation. The quality of the fit of a sufficiently complex machine learning model to the training data (i.e., the training error) is usually highly over-optimistic and not indicative of its later performance on unseen data. A proper validation for clinical practice thus comprises several steps [10], as follows:

1. Internal validation based on the initial discovery cohort. This can be achieved by setting parts of the data aside as an independent test set or, more frequently, via cross-validation. Cross-validation refers to a strategy in which subsequently a certain fraction (e.g., 10%) of the original data is left out for model testing and the remaining part is used for model training. The cross-validation procedure averages prediction performance over different test sets and thus reduces the variance in test set performance estimates. This is specifically relevant if the overall discovery cohort is not very large.
2. External validation based on an independent cohort. This is necessary to address the potential selection bias during the compilation of the discovery cohort.
3. Validation in a prospective clinical trial to demonstrate the benefit compared to standard of care.

The entire process is time-consuming and costly. Consequently, the number of clinically validated models is limited.

Overall, the current hype about machine learning and AI in healthcare has to be contrasted with a number of existing challenges, which can be summarized as:

- Insufficient prediction performance
- Challenges with model interpretation
- Challenges with validation and translation of stratification algorithms into clinical practice

These challenges lead to the fact that, in contrast to the very high expectations portrayed in the mainstream media, there exist only very few examples of machine learning-based solutions that impact clinical practice (see the examples mentioned above). In the following, we discuss some of these challenges in more detail and point to possible ways of addressing them today and in the future.

## What is possible today?

### Machine learning for personalized medicine

#### Defining better clinical endpoints

Many methodological as well as applied articles focus on simple yes/no decision tasks, e.g., disease progression / no disease progression or clinical trial endpoint met /not met. This is surprising, because machine learning research offers a comprehensive arsenal of techniques to address clinical endpoints beyond binary classification, such as, real valued, time-to-event, multi-class or multivariate outcomes. Models with binary outcomes can be appropriate in specific situations, but in many cases, an appropriate clinical outcome is more complex. For instance, the commonly used response criterion for rheumatoid arthritis, a debilitating autoimmune disease of the joints, is based on the DAS28 disease score [26], which ranges on a continuous scale from 0 to 10 and is often discretized into three consecutive levels (low, medium, high disease activity).

The DAS28 score itself combines four components in a nonlinear equation, namely the number of swollen joints, the number of tender joints, plasma levels of CRP protein, and an assessment of the patient’s global health as estimated by a physician. These components vary from discrete to continuous and from subjective, physician-dependent assessments to more objective measurements of biomarkers.

Another example is the prediction of response to anti-epileptic drug treatment. While at first glance overall seizure frequency reduction after a given number of weeks relative to baseline seems to be an appropriate endpoint in agreement to common practice in clinical trials, this choice in fact neglects the existence of different seizure types as well as the potential temporal modifications of these seizure types due to treatment. Thus, other and more complex (possibly multivariate) clinical endpoints might be necessary. We expect that a more careful choice of clinical endpoints as well as better technical monitoring capabilities (e.g., via mobile health applications and wearable sensors) will lead to more clinically useful prediction models in the future.

#### Defining appropriate model quality and performance measures

What makes a good model in personalized medicine? First, predictions must be accurate. As pointed out above, prediction accuracy must be assessed via a careful validation approach. Within such a validation procedure, it has to be decided how prediction performance will be measured. It appears that, in many studies, too much focus is given to standard, off-the-shelf metrics (e.g., area under the receiver operator characteristic curve), as compared to application-specific performance metrics. For instance, consider the case of predicting response to a first line therapy and assume that we can formulate this question as a classification task (responder vs. non-responder). Clearly, a perfectly accurate classifier is optimal. However, even a classifier that is mediocre with respect to overall accuracy might reliably identify those patients that will definitely not respond to the drug. The identified patients could immediately move on to a second line therapeutic and, thus, patient quality of life would improve and healthcare costs could be reduced. This example demonstrates the relevance of carefully defining appropriate prediction performance metrics.

However, prediction performance is only one aspect of judging the overall quality of a model. Another aspect is model stability, which reflects the degree to which a model (including variables selected by that model) remains the same if the training data is slightly changed. Model stability is a particular issue when working with gene expression data, where models trained on very different or even disjoint gene subsets can result in similar prediction performance regarding a given clinical endpoint, since highly correlated features can be substituted for each other [26]. Model stability should be routinely reported in addition to prediction performance.

Various methods have been developed for increasing the chance of obtaining a stable model during the development phase of a stratification algorithm. For example, inclusion of prior knowledge, such as biological networks and pathways, can enhance the stability and thus reproducibility of gene expression signatures [27–29]. Moreover, zero-sum regression [30] can be used to build classifiers that are less dependent on the employed omics platform (e.g., a specific microarray chip) [31], thus easing external validation, translation into clinical practice as well as long-term applicability of the model. We think that more frequent use of such methodology in conjunction with careful evaluation of model stability would lower the barrier for model transfer from discovery to external validation and finally to clinical application.

#### Tools for interpreting a machine learning model

As researchers collect and analyze increasingly larger sets of data, a greater number of sophisticated algorithms are employed to train predictive models. Some of the computational methods, in particular those based on deep learning techniques, are often criticized for being black boxes. Indeed, as the number of input features becomes large and the computational process more complex, understanding the reasons for obtaining a specific result is difficult, if not impossible. In many instances, for example, in the case of identification of disease markers, understanding the computational decision-making process leading to the selection of specific markers is, however, necessary and demanded by physicians. Using black-box models for medical decision-making is thus often considered to be problematic, leading to initiatives like the ‘right to an explanation’ law Article 22 of the General Data Protection Regulation propositioned by the European Union in April 2016/679. Similarly, in the process of drug development in pharmaceutical industry, regulatory agencies require transparency and supporting evidence of a molecular mechanism for the choice of specific biomarker panels.

**Fig. 4:**
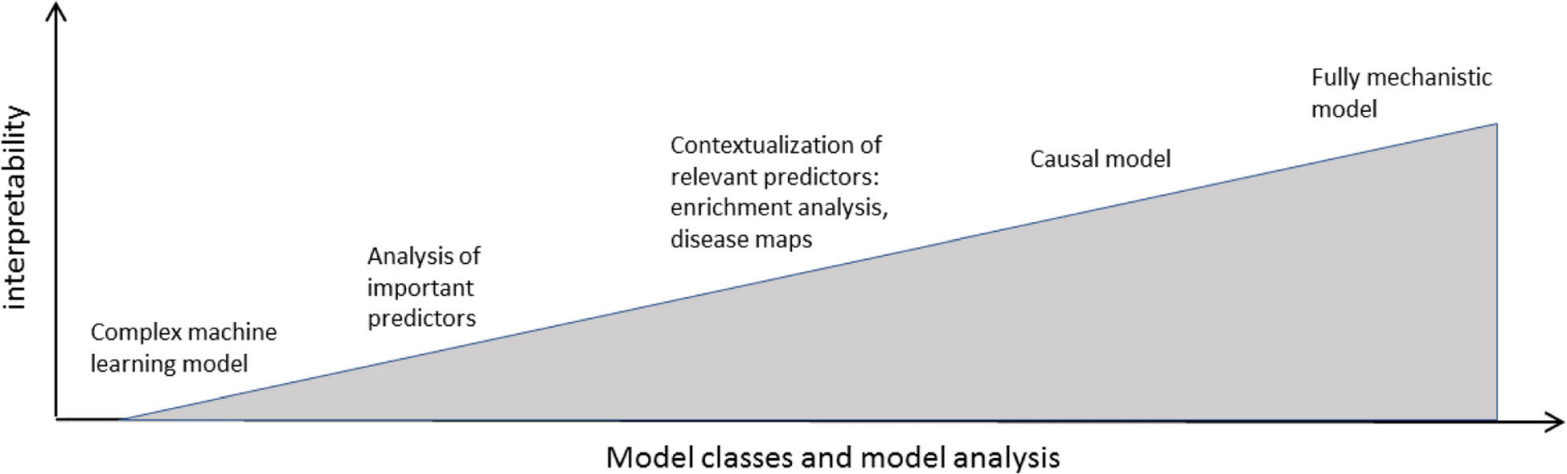
Different classes of machine learning models and their interpretability via model analysis

While usefulness of data-driven prediction is increasingly recognized, a key requirement for credibility of such solutions is thus the ability to interpret them in the context of current biomedical knowledge. It is important to understand that the concept of interpretability covers a spectrum (Fig. 4). At one end of the spectrum, there is a detailed understanding of the exact (biochemical) molecular and pathophysiological mechanisms that link a model with a defined clinical endpoint. Typically, this level of insight is rarely achievable due to lack of knowledge.

A less detailed level of understanding is that of total causal effects of a predictor regarding the clinical endpoint of interest. For example, in a randomized controlled clinical trial, any difference in outcomes between the two treatment groups is known to be caused by the treatment (since the groups are similar in all other respects due to the randomization). Thus, although one may not know exactly how the treatment affects the outcome, one knows that it does. Such statements about total causal effects are more difficult to obtain in a setting outside clinical trials, where purely observational data from untreated patients are collected (e.g., cross-sectional gene expression data). Nonetheless, computational approaches have significantly advanced in this field over recent years and, under certain assumptions and conditions, allow for estimating causal effects directly from observational data [32, 33].

At a lower level of interpretability, gene set and molecular network analysis methods [34, 35] can help to understand the biological sub-systems in which biomarkers selected by a machine learning algorithm are involved. There also exists a large body of literature on how to directly incorporate biological network information together with gene expression data into machine learning algorithms (see [28] for a review).

Recently, the concept of ‘disease maps’ has been developed as a community tool for bridging the gap between experimental biological and computational research [36]. A disease map is a visual, computer-tractable and standardized representation of literature-derived, disease-specific cause–effect relationships between genetic variants, genes, biological processes, clinical outcomes, or other entities of interest. Disease maps can be used to visualize prior knowledge and provide a platform that could help to understand predictors in a machine learning model in the context of disease pathogenesis, disease comorbidities and potential drug responses. A number of visual pathway editors, such as CellDesigner [37] and PathVisio [38], are used to display the content of a disease map and to offer tools for regular updating and deep annotation of knowledge repositories. In addition, dedicated tools such as MINERVA [39] and NaviCell [40] have been developed by the Disease Map community. At this point in time, disease maps are more knowledge management rather than simulation or modeling tools, although intensive efforts are underway to develop the next generation of disease maps that are useful for mathematical modelling and simulation and become an integral part of data interpretation pipelines.

The least detailed level of understanding of a complex machine learning algorithm is provided by the analysis of relative importance of variables with respect to model predictions. Relative variable importance can be calculated for a range of modern machine learning models (including deep learning techniques), but the level of insight depends on whether only few out of all variables have outstanding relevance and whether these variables can be contextualized with supporting evidence from the literature. It is also not clear a priori if such variables are only correlated with or perhaps also causal for the outcome of interest. Finally, inspecting most important variables may be less informative in the case of highly collinear dependencies among predictor variables such as, for example, in gene expression data.

In addition to the interpretation of predictors there is a need from a physician’s perspective to better understand model predictions and outputs for a given patient. One obvious way might be to display patients with similar characteristics. However, the result will depend on the exact mathematical definition of similarity. Moreover, clinical outcomes of most similar patients will, in general, not always coincide with the predictions made by complex machine learning models, which could result in misinterpretations. The same general concern applies to approaches, in which a complex machine learning model is approximated by a simpler one to enhance interpretability, for example, using a decision tree [41, 42].

### Data type-specific challenges and solutions

#### Real-world longitudinal data

Longitudinal EMR and claims data have received increasing interest in recent years within the field of personalized medicine [43, 44] since they provide a less biased view on patient trajectories than data from classical clinical trials, which are always subject to certain inclusion and exclusion criteria [45]. Specifically in the United States, a whole industry has grown to collect, annotate, and mine real-world longitudinal data (https://cancerlinq.org/about, https://truvenhealth.com/). The recent US$1.9 billion acquisition of Flatiron Health by the pharma company Roche (https://www.roche.com/media/store/releases/med-cor-2018-02-15.htm) marks the potential that is seen by industrial decision-makers in the context of drug development, pharmacovigilance, label expansion, and post-marketing analysis [45, 46].

Longitudinal real-world data pose specific challenges for training and validation of predictive models. Within the analysis of clinical real-world databases (e.g., Clinical Practice Research Datalink; https://www.cprd.com/home/) patients for a study cohort are typically selected based on a specified index date or event, which is often difficult to define and thus leaves room for different choices. Since the maximal observation horizon in real-world databases is often limited to a certain number of years (e.g., due to budget restrictions), some patients are longer observed than others. Specifically, claims data may contain gaps (e.g., due to periods of unemployment of patients) and the exact date of a diagnosis, prescription, or medical procedure cannot be uniquely determined. It is not always clear for the treating physician which ICD diagnosis codes to choose, and this leaves room for optimization with respect to financial outcomes. In addition, EMRs require natural language preprocessing via text mining, which is a difficult and potentially error-prone procedure in itself. In conclusion, development of a predictive model for personalized medicine based on real-world clinical data thus remains a non-trivial challenge.

Classically, validation of a predictive model relies on an appropriate experimental design and randomization. Real-world data often limits the options available for rigorous validation. Classical strategies, such as carefully crafted cross-validation schemes, can offer reliable validation, but they might be tricky to design, and the limits of such retrospective validation must be properly understood. Another option is the use of different time windows where only retrospective data up to a given date is used to develop a model, which is then used on the data available after this date. Such a setup can be close to an actual prospective evaluation, although the risk for biases is larger. Another option is to consider such analyses as only generating hypotheses, which are then followed up in a more classical fashion by setting up a carefully designed observational study manifesting the final validation. A more speculative possibility is the adaptation of so-called A/B testing techniques that are common in web development and software engineering [47]. This would entail randomization of patients for therapeutic options directly in the real-world environment. While such a setting is probably not feasible for drug development, it may be applicable to determine the efficacy of interventions in a real-world setting or to determine the right patient population for a given intervention.

#### Multi-modal patient data

There is an increasing availability of multi-scale, multi-modal longitudinal patient data. Examples include the Alzheimer’s Disease Neuroimaging Initiative (http://adni.loni.usc.edu/) (omics, neuro-imaging, longitudinal clinical data), the Parkinson’s Progression Markers Initiative (http://www.ppmi-info.org/) (omics, neuro-imaging, longitudinal clinical data), the All-of-Us Cohort (https://allofus.nih.gov/) (omics, behavioral, EMRs, environmental data), the GENIE project (http://www.aacr.org/Research/Research/Pages/aacr-project-genie.aspx#.WvqxOPmLTmE) (genomic and longitudinal real-world clinical data) and, specifically for multi-omics, the NCI’s Genomic Data Commons [48]. Multi-modal data provide unique opportunities for personalized medicine because they allow for capturing and understanding different dimensions of a patient. This aspect is in turn widely believed to be key for enhancing the prediction performance of stratification algorithms up to a level that is useful for clinical practice. Accordingly, there has been a lot of work in methods that combine data from different (omics-) modalities, see [49] for a review.

**Fig. 5:**
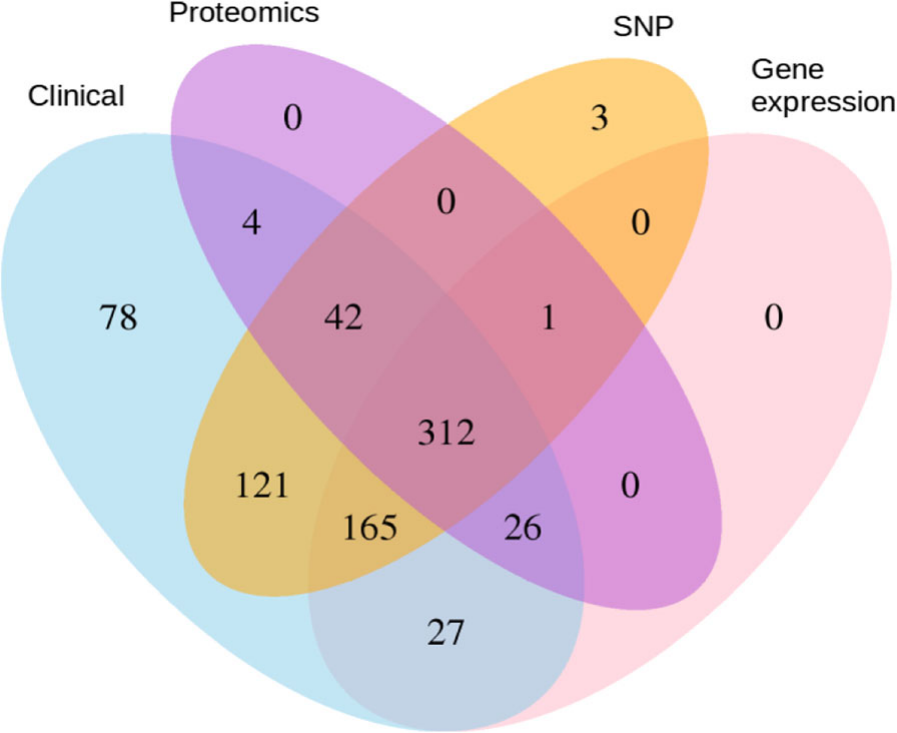
Overlap of different omics data entities and clinical data in the AddNeuroMed Alzheimer’s Disease cohort from EMIF-AD (http://www.emif.eu/about/emif-ad). Numbers refer to patients, for which a particular data modality is available

A major bottleneck in current studies collecting multiple data modalities of clinical cohorts is posed by the fact that different studies are often performed on cohorts of different patients and different experimental approaches are used across studies (see Fig. 5 for an example). As consequence, data from different studies becomes difficult or even impossible to integrate into a joint machine learning model. Several strategies are possible to reduce this problem in the future. A first strategy is to perform systematic multi-modal data assessment of each individual in a clinically rigorously characterized cohort, including longitudinal clinical and omics follow-up. In the more classical clinical setting, the success of the Framingham Heart Study (https://www.framinghamheartstudy.org/) comes to mind, which is a long-term study about risk factors for cardiovascular diseases running since 1948. While, in the future, we will analyze larger and larger volumes of real-world data, we should be aware of the limitations of such data (interoperability of data from different sources, non-systematically collected data, measurement quality, inconsistencies and errors, etc.). Rigorous multi-modal observational studies are essential for establishing reliable baselines for the development of real-world models. Ideally, multi-modal data would be collected longitudinally in regular intervals for all subjects. While this has been achieved for individual studies [50], for practical and economic reasons, this is likely to be limited to a small number of cohorts. A second approach is to have some overlap among patients across different cohorts. Statistical methods and machine learning can then be used to ‘tie’ different datasets together. A third approach is to collect a joint modality (such as standardized clinical data or biomarkers) across different studies. This joint modality again makes it possible to tie together different datasets. It must be stressed that this problem of disconnected cohorts is currently a major obstacle for leveraging multi-omics data.

It should be emphasized that, ideally, multi-modal, multi-omics data should be considered in conjunction with longitudinal clinical data. Despite of the examples mentioned above (Alzheimer’s Disease Neuroimaging Initiative, Parkinson’s Progression Markers Initiative, All-of-Us Cohort) we are currently just in the beginning of performing corresponding studies more systematically. The combination of multi-omics with real-world longitudinal data from clinical practice (e.g., EMRs) and mobile health applications marks a further potential for personalized medicine in the future. The GENIE project is an important step into this direction.

### Translating stratification algorithms into clinical practice

The ability to accelerate innovation in patient treatment is linked to our ability to translate increasingly complex and multi-modal stratification algorithms from discovery to validation. Stratification in clinical application means assigning treatment specifications to a particular patient, which may include type, dosage, time point, access to the treatment, and other pharmacological aspects. The validation of such algorithms is usually performed via internal validation (cross-validation), external validation (using a separate patient cohort), and prospective clinical trials compared to the standard of care [10] (http://www.agendia.com/healthcare-professionals/the-mindact-trial/). Proper validation constitutes a requirement for translating these methods to settings in which they can generate impact on patient outcomes. In addition to classic healthcare providers, such as hospitals and general practitioner, mobile health applications and wearable sensors might play an increasing role in the future. As described earlier, integrating multi-modal data is key for gaining new insights and lies also at the heart of stratifying patients for diagnostic, predictive, or prognostic purposes. However, considerable barriers exist regarding the integration of similar data from different cohorts, normalization of data across measurement platforms, and the ability to process very large volumes of data in appropriate systems close to or within the clinical infrastructure remains limited. Strictly controlled cloud services, which appropriately protect patient data, could be an approach to alleviating this limitation [51]. At this point it might be possible to learn from organizations that today handle large scale real-world clinical data (mostly in the US). However, their approaches may have to be adapted to the legal environments in each specific country.

At present, translation of algorithms for patient stratification into clinical practice is also difficult due to regulatory aspects. Prospective clinical trials required for approval of diagnostic tools by regulatory agencies are very costly and the challenges for finding sponsors are high. One possibility of lowering the associated barriers might be to perform a stepwise approach with initial pilot studies to exemplify the value that can be gained for patients, healthcare sustainability, translational science, and economic efficiency. Such projects would need to showcase the principle value of patient stratification. Moreover, they could provide meaningful insights into disease biology (via biomarkers). These outcomes should ideally be measured longitudinally after machine learning-based stratification and thus provide a feedback loop that helps improve the stratification algorithm.

A commonly stated myth is that health innovation is based on the paradigm of build-and-freeze (https://www.theatlantic.com/technology/archive/2017/10/algorithms-future-of-health-care/543825/), which means that software is built, frozen, and then tested in unchanged form for its lifetime. However, development of better stratification algorithms will require a more seamless updating scheme. There have been interesting developments in recent years in terms of regulation and risk management for continuous learning systems. An example of such a development is the Digital Health Software Precertification (Pre-Cert) Program (https://www.fda.gov/MedicalDevices/DigitalHealth/DigitalHealthPreCertProgram/Default.htm) launched recently by the FDA. PreCert aims at learning and adapting its key elements based on the effectiveness of the program. In addition, Clinical Laboratory Improvement Amendments (CLIA; https://www.fda.gov/MedicalDevices/DeviceRegulationandGuidance/IVDRegulatoryAssistance/ucm124105.htm) labs provide a template for how health-related software tools developed to inform precision medicine can be validated in a clear and transparent manner as the tool is continually updated. CLIA labs are certified labs that go through a process of regular certifications monitored by the FDA and other regulatory agencies in the US. These labs are required to follow approved and documented Standard Operation Procedures. They can use medical devices, which can include software for diagnostics, given that they employ such Standard Operation Procedures and waive the certification process (https://wwwn.cdc.gov/clia/Resources/WaivedTests/default.aspx). Most importantly, the developer of the tool can update the software. The CLIA labs are independent in deciding whether they will re-validate the software and can adopt a strategy that best serves the technological pace of the software and their clinical needs with respect to increased capabilities or better performance. For instance, a lab may decide to validate only major version releases, such as going from version 1.x to 2.0, and have minor version releases included on the fly.

The vision of precision medicine is to provide the right intervention to the right patient, at the right time and dose. The described approaches, based on iterative feedback between the developers and the clinical end users, could increase our ability to adapt stratification algorithms better to new insights in disease biology, access to new molecular data, and changes in clinical settings. This has been a challenge with promising predictive models often failing validation in independent studies. Real-world longitudinal data from clinical practice and data collected through wearables or other means of participatory data collection cannot only widen the spectrum of possible data sources to build new stratification algorithms [52, 53], but they may also be partially included in clinical trials for validation purposes of stratification algorithms.

## What could be possible tomorrow?

### Novel approaches to better link prediction algorithms with biomedical knowledge

As discussed earlier, challenges with the interpretation of complex machine learning models are one of the important bottlenecks for applying personalized medicine more widely. Innovative software solutions are needed to better put complex machine learning models and their outputs into the context of computationally accessible knowledge of human pathophysiology.

While the current standard is to map the most relevant molecular features in a machine learning model onto biological pathways, this approach could be further enhanced to make machine learning-based decisions interpretable by clinicians. In the future, one might imagine software systems that automatically collect information on each variable from various databases and publications (e.g., via text mining). Such tools could eventually even compose entire reports (including supporting texts and figures of disease maps) for each individual feature in a machine learning model. Such reports could thus automatically contextualize each variable with the multitude of available biomedical knowledge in a fully interactive fashion. The physician could zoom and filter specific aspects of a model on demand.

Another idea is to visualize entire patient trajectories (originating, for example, from longitudinal clinical trials, real-world clinical or behavioral data) within interactive ‘disease landscapes’ (essentially low-dimensional data projections). Such a tool could help physicians to understand disease development over time. Taking the patient’s history into account will allow clinicians to visualize and interpret the speed and severity of disease progression. Individual patient trajectories could then be followed and compared to determine, for example, which intervention is appropriate for which patient and at what time [54]. Similar concepts have been developed in other contexts, e.g. for estimating the in-vivo fitness landscape experienced by HIV-1 under drug selective pressure [55].

The development of such methods and software systems will be a major effort and will likely require a substantial text analytical and software engineering component. However, such systems could greatly facilitate the communication between computational scientists and physicians and help make complex machine learning models more interpretable.

### Going from ‘what’ to ‘why’ – towards better interpretable modeling approaches

#### Causal models

Machine learning models are typically neither mechanistic nor causal. They largely capture (non-linear) correlations between predictor variables and clinical outcomes and are thus often criticized for being black boxes. The main advantage of modern machine learning approaches is that they neither require a detailed prior understanding of cause–effect relationships nor of detailed mechanisms. The main limitation is the difficulty to interpret them (see previous Section). A major question thus relates to how far machine learning methods could evolve into more causal models in the future.

Causal graphical models (causal Bayesian networks in particular) constitute an established framework for causal reasoning [56]. They provide a compact mathematical and visual representation of a multivariate distribution, and more importantly, they allow to make predictions of the system under unseen interventions (e.g. a new treatment or a gene knockout). Under appropriate assumptions, causal graphical models can be learned from observational data [57–59]. In doing so, it is also possible to incorporate background knowledge or to allow for hidden or unmeasured confounders. We refer to [60] for a review paper.

Causal graph learning methods may play an increasingly important role in the future in identifying predictor variables with causal influence on clinical outcomes [61] and may thus help to move towards a causal interpretation of predictor variables in a machine learning model [62]. However, there are non-trivial challenges that need to be addressed, such as dealing with violations of assumptions, high computational costs and non-linear relationships [63].

#### Hybrid machine learning and mechanistic models

Despite the increasing availability of massive datasets, the predictive power of most of the available disease models does not yet satisfy the requirements for clinical practice. One of the reasons is that, in principle, predictive disease models must cover all relevant biotic and abiotic mechanisms driving disease progression in individual patients. Although the primary disease-driving mechanisms are often aberrations at the molecular level, such as mutations in the genome, disease progression is affected by the robustness of the overall system. However, biological systems have established a multitude of repair mechanisms to compensate for the effects of molecular aberrations, thus introducing feedback loops and non-linear interactions into the system [64]. Overall, disease progression is a process affected by a multitude of highly diverse mechanisms across biological hierarchies, which are differently expressed in individual patients.

Thus, a disease model, designed for applications in precision medicine in clinics, must in principle integrate three conceptual layers:

- A core disease model (CDM) represents only the known intra- and inter-cellular processes that are the key drivers of the disease in an average patient.
- The CDM must be adapted to the individual patient and their specific medical history and environment, such as genetic variations, co-morbidities or physiology, by environment adaption models (EAM). The EAM must provide an individualization of the parameters controlling the CDM, eventually combined with an individualized re-structuring of the CDM, e.g., by adding or dropping biological mechanisms that are relevant only in specific patient populations.
- Monitoring models must be developed to describe how clinically accessible outcome measurements representing the disease evolution are linked to the CDM.

Today, fully mechanistic models exist for a series of disease-driving core processes at the molecular and cell population level [65]. However, broader application of mechanistic modelling to implement the CDM for complex diseases is hampered by insufficient knowledge of the interaction of the core disease-driving mechanisms across scales. Even worse, the relevant mechanisms for EAM and monitoring models are almost never completely known. Altogether, it thus seems unlikely that fully mechanistic models will play a dominant role in personalized medicine in the near future.

While machine learning models are not harmed by insufficient biomedical knowledge, they are often criticized for their black-box character. Hybrid modelling, also named grey-box or semi-parametric modelling, is an integrative approach combining available mechanistic and machine learning-based sub-models into a joint computational network. The nodes represent model components and the edges their interaction. First combinations of mechanistic and data-driven models have been developed for chemical and biotech process modelling [66, 67]. For example, neural networks have been used to compensate the systematic errors of insufficient mechanistic models, to estimate unobservable parameters in mechanistic models from observable data, or to estimate the interaction between different mechanistic sub-models [68, 69].

A further successful example of hybrid modeling comprises learning the drug mechanism of action from data [70, 71]. Hybrid models may thus be a way to combine the positive aspects of fully mechanistic and purely data-driven machine learning models. First showcases have demonstrated the potential, but more successful applications are needed. Moreover, a deeper understanding of the theoretical capabilities of hybrid models as well as their limitations is necessary.

### Controlling critical transitions in patient trajectories

One of the key objectives of personalized medicine is predicting the risk of an individual person to develop a certain disease or, if the disease has already developed, to predict the most suitable therapy. This also includes predicting the likely course of disease progression. Disease trajectories entail all the hallmarks of a complex system. In this sense, modeling disease trajectories is not fundamentally different from attempts to model and simulate other complex systems such as the climatological, ecological, economic or social systems. In many of these highly nonlinear, complex systems with thousands or millions of components, involving redundant and intertwined feedback relations, so called critical transitions or catastrophic shifts can be observed. Such transitions are defined by critical thresholds, sometimes called tipping points at which a system transitions abruptly from one state to another, seem to exist. However, in many of these cases, critical transitions are extremely difficult to predict in advance.

For certain diseases, we believe that the concept of critical transitions might also be applicable in the context of personalized medicine. Tipping points are often observed during the course of acute or chronic disease development. The ability to predict a critical transition of a developing disease before it really happens would be highly desirable and provide very valuable pre-disease biomarkers.

Recently, Liu et al. [72] used gene expression analysis to develop the concept of dynamic network biomarkers, where higher-order statistical information is used to identify upcoming tipping points. The idea is that, during the disease trajectory, a subset of genes starts to fluctuate and leads to a destabilization of a (possibly high-dimensional) attractor state. By measuring the changes in gene correlation in addition to changes in the variation of gene expression, a quantitative index was proposed as an early warning signal for a critical transition.

### Towards an evolutionary understanding of human disease

From a broader perspective, evolutionary principles could help to improve our understanding of human disease [73]. Evolutionarily conserved control genes are probably highly relevant for the proper functioning of molecular pathways [74], and evolutionary history of human disease genes reveals phenotypic connections and comorbidities among some diseases [75]. We are now at the verge of reconstructing the molecular and cellular circuitry of embryogenesis [76]. In addition, whole-genome next-generation sequencing efforts of hundreds of thousands and soon Millions of patients with common and rare diseases provide us with a rich genotype–phenotype landscape underlying the development and manifestation of human diseases. Such data provides interesting opportunities to better understand the influence of genomic variants on evolutionarily conserved genomic regions and molecular networks in the context of human diseases.

Evolutionary conservation might be relevant for constraining models and simulating human diseases. Biologically possible and plausible disease trajectories are likely limited by the topological and dynamic upper and lower bounds that are set by the evolutionary history of a disease network. A key challenge for personalized medicine is to come up with a mechanistic explanation of an individual’s disease development. We need to understand the effects of genetic variation on the resulting phenotypic variation. This requires close cooperation between disciplines striving for an integration of the concepts of ontogeny and phylogeny. Human diseases must be seen in the light of evolution and models of human diseases need to integrate data, information, and knowledge from developmental biology and embryology.

## Conclusions

In the era of growing data volumes and ever shrinking costs for data generation, storage, and computation, personalized medicine comes with high promises, which can only be realized with the help of advanced algorithms from data science, particularly machine learning. Modern machine learning algorithms have the potential of integrating multi-scale, multi-modal, and longitudinal patient data to make relatively accurate predictions, which, in some examples, may even exceed human performance [21]. Large commercial players that are now entering the field of medicine underline the potential that is widely seen for computational solutions.

However, the current hype around AI and machine learning must be contrasted with reality. While many prediction algorithms for patient stratification have been published over the last decade, only very few approaches have reached clinical practice so far. Major existing bottlenecks discussed in this paper include the (1) lack of sufficient prediction performance due to a lack of signals in the employed data; (2) challenges with model stability and interpretation; (3) a lack of validation of stratification algorithm via prospective clinical trials, which demonstrate benefit compared to standard of care; and (4) general difficulties to implement a continuous maintenance and updating scheme for decision support systems.

In addition, general concerns around data privacy as well as ethical and legal aspects must not be overlooked. To overcome these hurdles, an interdisciplinary effort including computational scientists, physicians, patient advocates, regulatory agencies, and health insurance providers is required in the context of a ‘learning healthcare system’ (http://www.learninghealthcareproject.org/section/background/learning-healthcare-system). There is a need to better manage the (partially unrealistic) expectations and concerns about data science and AI-based solutions.

In parallel, computational methods must advance in order to provide direct benefit to clinical practice. Current algorithms are far from being able to recommend the right treatment at the right time and dose for each patient. Steps that bring us closer to this goal could be (1) innovative software tools that better link knowledge with machine learning-based predictions from multi-scale, multi-modal, and longitudinal data; (2) innovative modeling approaches, such as causal inference techniques and hybrid modeling, which go beyond typical state-of-the-art machine learning; and (3) new computational modeling approaches that allow us to identify critical transitions in a patient’s medical trajectory.

More speculatively, a broader understanding of human disease, incorporating findings from basic research and evolutionary studies, might help the creation of entirely new concepts for simulating human diseases and predicting optimal intervention points. Overall, the ambition of research towards personalized medicine should be to move from a system analysis perspective (such as in molecular biology) to a system control view that allows for the planning of optimal medical interventions at the right time and dose on an individualized basis. Novel computational modeling approaches that go beyond the current machine learning methodology may play an increasing role for that purpose.

In this context, it must be emphasized that no algorithm is meant to replace a physician. Rather, the idea is to provide them a tool at hand, which supports their decisions based on objective, data-driven criteria and the wealth of available biomedical knowledge.

## Abbreviations

AI: Artificial Intelligence
CDM: core disease model
CLIA: Clinical Laboratory Improvement Amendments
EAM: environment adaption model
EMR: electronic medical record
FDA: Food and Drug Administration
